# Comparative Magnitude of Cross-Strain Conservation of HIV Variable Loop Neutralization Epitopes

**DOI:** 10.1371/journal.pone.0015994

**Published:** 2010-12-29

**Authors:** James Swetnam, Evgeny Shmelkov, Susan Zolla-Pazner, Timothy Cardozo

**Affiliations:** 1 Department of Pharmacology, New York University School of Medicine, New York, New York, United States of America; 2 Department of Pathology, New York University School of Medicine, New York, New York, United States of America; 3 New York Veterans Affairs Medical Center, New York, New York, United States of America; University of Rochester, United States of America

## Abstract

Although the sequence variable loops of the human immunodeficiency virus' (HIV-1) surface envelope glycoprotein (gp120) can exhibit good immunogenicity, characterizing conserved (invariant) cross-strain neutralization epitopes within these loops has proven difficult. We recently developed a method to derive sensitive and specific signature motifs for the three-dimensional (3D) shapes of the HIV-1 neutralization epitopes in the third variable (V3) loop of gp120 that are recognized by human monoclonal antibodies (mAbs). We used the signature motif method to estimate the conservation of these epitopes across circulating worldwide HIV-1 strains. The epitope targeted by the anti-V3 loop neutralizing mAb 3074 is present in 87% of circulating strains, distributed nearly evenly among all subtypes. The results for other anti-V3 Abs are: 3791, present in 63% of primarily non-B subtypes; 2219, present in 56% of strains across all subtypes; 2557, present in 52% across all subtypes; 447-52D, present in 11% of primarily subtype B strains; 537-10D, present in 9% of primarily subtype B strains; and 268-D, present in 5% of primarily subtype B strains. The estimates correlate with *in vitro* tests of these mAbs against diverse viral panels. The mAb 3074 thus targets an epitope that is nearly completely conserved among circulating HIV-1 strains, demonstrating the presence of an invariant structure hidden in the dynamic and sequence-variable V3 loop in gp120. Since some variable loop regions are naturally immunogenic, designing immunogens to mimic their conserved epitopes may be a promising vaccine discovery approach. Our results suggest one way to quantify and compare the magnitude of the conservation.

## Introduction

The gp120 surface envelope glycoprotein of HIV-1 (HIV) is the primary target for antibodies that neutralize HIV infection *in vitro* [1], and passive transfer of HIV gp120-specific antibodies confers protection from HIV and chimeric simian-human immune deficiency virus (SHIV) challenge in several animal models [2]–[8]. Furthermore, some highly sequence variable regions of gp120 are strongly immunogenic [9]–[13], and are documented to make up a proportion of the serum neutralizing antibody response to immunization with gp120 [14]. Unfortunately, extraordinary antigenic diversity is a hallmark of the HIV Group M strains that are causing the acquired immune deficiency syndrome (AIDS) pandemic, and this diversity is especially prominent in the sequence variable regions, including the V3 loop [15].

One of the most productive approaches to interrogating the antigenic diversity of HIV viruses for conserved shapes that may be immunologically targeted is to isolate monoclonal antibodies from HIV-infected human subjects and study their specificities, specifically their ability to neutralize *in vitro* infection by diverse HIV viruses. For example, two constant regions in gp120 and gp41, the CD4 binding site (CD4bs) of gp120 and the membrane proximal external region (MPER) of gp41, have been the subject of much study, primarily because they are targeted by three broadly neutralizing human mAbs, b12, 2F5 and 4E10. However, the breadth, potency and activities of the hundreds of variable-loop-specific monoclonal antibodies (mAbs) isolated from HIV-positive subjects varies widely, as expected given the antigenic diversity in these regions [16], [17]. Many such mAbs are non-neutralizing, as expected. Among those with neutralizing activities, many are strain- or type-specific [18], while others display neutralizing activity across subtypes [19]–[23], including anti-V3 loop mAbs, which show some of the broadest patterns [24], [25]. Accordingly, the variable loops, and the V3 loop in particular, have been considered improbable targets for vaccine-induced protective antibodies [26], despite the observed immunogenicity advantages and despite extensive evidence that there is a great deal of immunologic cross-reactivity among V3 loops from diverse strains and clades [27]–[29].

Antibody epitopes are traditionally defined immunochemically via epitope-mapping experiments using nested sets of linear peptides or libraries of mutated antigens, but this approach cannot be translated to easily measure the conservation of epitopes across the informatics of the large number of circulating HIV-1 strains. We previously approached the problem from a purely 3D structural point of view in order to achieve the translation of vaccine-relevant molecular features to clinically relevant informatics observations (viral sequences). In the 3D structural view, antibodies recognize only a small 3D geometric surface, or “knob” on the surface of an antigen that is evident in the crystal structure of an Ab:epitope complex. We showed we could work back to the viral sequence from the 3D structure of the Ab:epitope complex and define a specific signature motif for the epitope recognized by the mAb that is evident simply by viewing any HIV-1 gp120 sequence [30]. This prototype method was applied to mAbs 447-52D and 2219 from the crystal structures of these mAbs complexed with V3 loop peptides and demonstrated that pseudoviruses with sequences containing the epitope signature motifs are highly sensitive to the mAbs targeting that motif, while those lacking the motifs were poorly sensitive to neutralization. The prior results demonstrated that the signature motifs were highly sensitive and specific for neutralization epitopes, and that the motifs could be used to search the HIV compendium to estimate the conservation of the 2219 and 447-52D epitopes across all circulating HIV-1 strains. One important aspect of this finding was that the motifs were sensitive and specific for epitopes displayed by viruses tested *in vitro*, so the use of the motifs incorporates the wide range of flexibilities exhibited by different V3 loop sequences.

Additional structures of novel anti-V3 mAbs isolated from HIV-positive patients have recently become available, including structures for 2557 [31], 3791 (unpublished data), 3074 [31], 268 [31] and 537-10D [32], along with more recent WHO data on the subtype classification of circulating HIV-1 strains [33]. We used these new data to estimate the range of conservation of neutralization epitopes targeted by a diversity of anti-V3 mAbs.

## Materials and Methods

3D-structure-based sequence motifs for neutralization epitopes were derived as previously described [30], including neutralization assays of diverse chimeric pseudoviruses demonstrating the specificity of each mAb for viruses displaying their cognate motif (data not shown). For mAbs 3074 [31], 2557 [31], 3791 (X-P Kong, personal communication), 537 [32], and 268 [31], the motifs were derived from crystal structures of these mAbs complexed with various V3 loop peptides (Table 1).

**Table 1:** Sequence Motifs for Epitopes Targeted by Individual anti-V3 mAbs Derived from 3D Structures of mAbs:V3 Complexes.

mAb	Infecting Donor HIV Subtype	V3 Loop Viral Peptide Bound to mAb in Crystallographic Complex (Superscript indicates V3 loop numbering from beginning to end of epitope area bound by mAb in the structure)	Sequence Motif for Epitope (Superscript indicates V3 loop numbering)
268-D	B	MN (R^10^KRIHIGPGR^18^AFA)	^10^[R,K]xx[H,R]xxPxR^18^
447-52D	B	MN (KRIHIG^16^PGR^18^A)	^16^PxR^18^
537-10D	B	MN (^9^RKRIHIGPGR^18^AFYAT)	^9^Rxxxx[I,M]xPxR^18^
2219	B	MN (K^9^RKRIHIGPGRAFY^21^TTKNA)	^9^RKx[I,V]xxxxxxxx[Y,F]^21^
2557	CRF02_AG	NY5 (TK^10^KGIAIGPGRTLY^21^)	^10^Kx[I,V]xxxxxxxxY^21^
3074	CRF02_AG	VI13041 (ATRKGIH^14^IGPGRAF^20^YA)	^14^[I,L,M]xPxxx[F,W]^20^
3791	C	CA7 (R^11^SIRIGSGQ^18^TSYATGA)	^11^SxRIxxxQ^18^

Infecting Donor HIV Subtype indicates the subtype of the virus infecting the patient from which the mAb was isolated. V3 loop numbering is as described in the Methods. “x” in the sequence motifs indicates that any amino acid may occupy that position.

**Figure 1:**
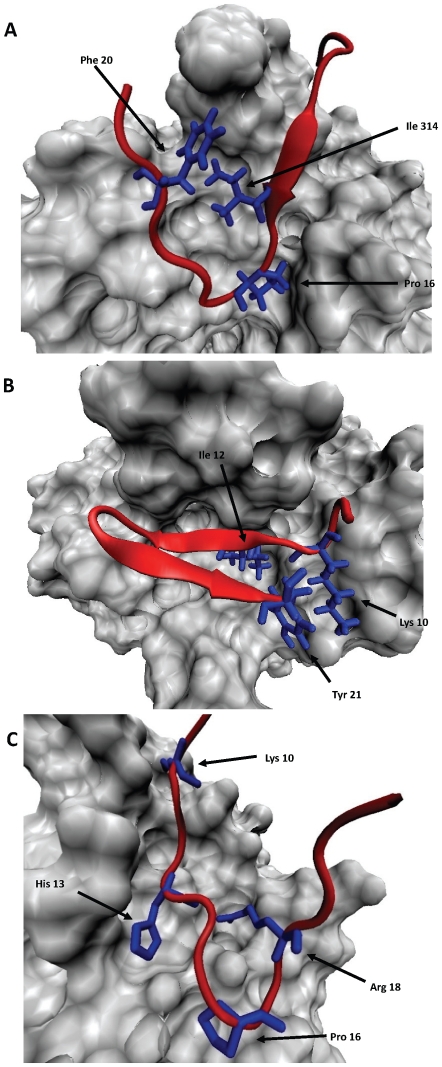
mAb 3074, 2557, and 268-D bound to V3 loop peptides. (A) Structure of mAb 3074 (grey molecular surface) bound to a peptide (red ribbon and blue stick depiction) with the same sequence as the crown of the V3 loop of subtype AG isolate VI191. (B) Structure of mAb 2557 (grey molecular surface) bound to a peptide (red ribbon and blue stick depiction) with the same sequence as the crown of the V3 loop of subtype B isolate NY5. (C) Structure of mAb 268-D (grey molecular surface) bound to a peptide (red ribbon and blue stick depiction) with the same sequence as the crown of the V3 loop of subtype B isolate MN. The side-chains of the V3 peptide that are buried in the molecular surface of the mAb are colored blue and labeled.

Briefly, a signature motif for an epitope is generated via examination of sequence-specific atomic contacts in a co-crystal structure between the monoclonal antibody (mAb) and a bound peptide's side chain atoms (Examples for mAbs 3074, 2557 and 268-D are shown in Figure 1). Only contacts in which the side-chain is largely buried in a pocket on the mAb molecular surface are included in the motif, as these were shown in the previous study [30] to be neutralization-specific. Any mAb contacts with peptide main-chain atoms are not included in the motif, as these are not sequence (side-chain) specific, and viral escape mutations that change the side chain will not necessarily alter the backbone or the mAb interaction. Side-chain contacting, but not buried in, the antibody molecular surface were shown by the previous study not to correlate with neutralization sensitivity (although they may still influence antibody binding). Where a strong (buried) side-chain contact is observed, an amino acid alphabetical letter is added to the signature motif. For example, the constraint “H13” would indicate that a His at position 13 is a necessary part of the neutralization epitope. Each motif was refined further by considering which amino acid side chains could substitute energetically at each key side-chain-buried contact locations identified as part of the motif. Only side chain substitutions occurring naturally in the viral population in more than 4% of recorded viruses were sampled at each position (data for positional variation was extracted from the Los Alamos National Laboratory (LANL) HIV Sequence Database, February, 2010, http://hiv.lanl.gov). Within a crystal structure of the Ab:V3-peptide complex the side chain in question was energetically minimized and the final energy recorded. The side chain was then *in silico* modified to a new side chain and energetically minimized and the new energy recorded. If the energy score of the substitution was within 1.5 of the native side-chain, the substitution was considered as energetically silent (non-disruptive). Such energetically silent substitutions were included in the motif even though they were not derived directly from the crystal structure.

For clarity, we used standard V3 loop numbering for the most common length of 35 residues of the V3 loop throughout this study. These numbers correspond to the standard HXB2 coordinate system as follows: HXB2 amino acid 296 is the same cysteine as position number 1 of the standard V3 loop; HXB2 amino acid 315 is the same as position 18 of the standard V3 loop.

Starting from these structure-based signature sequence motifs, the estimates of worldwide epitope coverage were determined using the steps described in our previously published method [30], modified with the latest 2004 epidemiological and sequence data available from Hemlaar, et. al. [33], while previously we used data derived by Osmanov et al. in 2002 [34]. Hemlaar et. al. collected three times as many samples as Osmanov et. al., included data from more countries, and collected data from more than one genome segment for subtyping, which provides valuable insight into genetic recombination in HIV. We also used an updated version of the LANL HIV Sequence Database (February, 2010, http://hiv.lanl.gov) for the results reported in this study.

## Results

**Figure 2:**
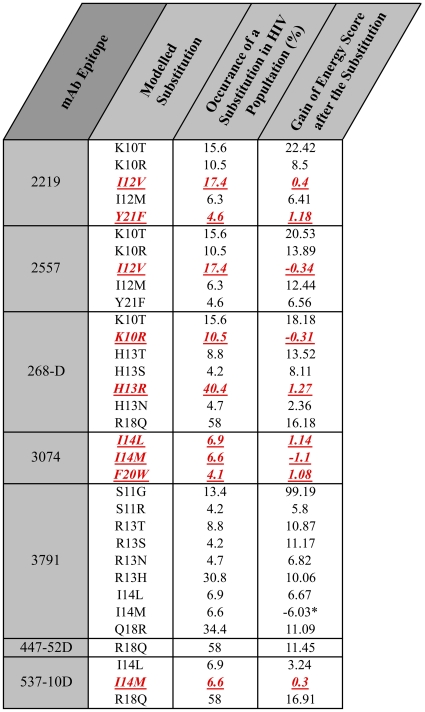
Results of the *in silico* modeling of amino acid substitutions in V3 loop epitopes. For each mAb epitope resolved crystallographically, *in silico* substitutions were made to the structure of the complex, the perturbation of the change was minimized conformationally and the change in energy of the complex was calculated as described in the Methods (right column). Only amino acid substitutions occurring naturally in the viral population in more than 4% at each position were considered in the study. Red colored values indicate energetically silent or non-disruptive substitutions that retained a similar mAb contact and which were then added to the motif describing the epitope. *One change resulted in energetic improvement but a conformational change such that contact with the mAb was lost, thus not contributing to the motif.

The 3D contact surface for each V3 peptide with each neutralizing mAb was carefully examined to derive the signature motif for the neutralization epitope as described in the Methods. In each case, V3 loop amino acid positions making contact with the mAb only through their backbone atoms were deemed tolerant positions and not included in the sequence motif, whereas a contact between the V3 peptide and the mAb characterized by partial burial of the V3 side chain into the molecular surface of the mAb was deemed an intolerant position that corresponds to a defined amino acid letter in the sequence motif. Examples of the key buried V3 loop side chains from this analysis are shown in blue stick depiction in Figure 1 for three of the antibodies 3074, 2557 and 268-D. The 3074:V3 molecular interface is characterized by one hydrophobic side-chain (Ile14) from the N-terminal β-strand and one hydrophobic side-chain from the C-terminal β-strand (Phe 20) of the V3 loop crown clustered together at the center of a twisted β-hairpin and buried in a central hydrophobic pockets on the mAb surface (Figure 1A). The proline at position 16 of the V3 loop is also buried in the mAb molecular surface. These three side chains cannot vary significantly without disrupting the complex, but side-chains at all other positions can vary without significantly disrupting the complex. Thus, only viral escape mutations at I14, P16 and F20 can disrupt neutralization mediated by 3074, while sequence variation elsewhere in the V3 loop leaves the interaction unaffected. The sequence motif for the predicted epitope targeted by mAb 3074 is therefore “I^14^-x-P^16^-x-x-x-F^20^” using V3 loop numbering. Inspection of the bioinformatics variation patterns at the positions 14, 16 and 20 revealed that besides Ile, Pro and Phe, observed at these positions in the crystal structure, Leu and Met at the position 14 and Trp at the position 20 occurred in more than 4% of recorded viruses. Modeling showed that Leu and Met substitutions for Ile at position 14 and Trp substitution for Phe at position 20 are energetically silent (see Methods and Figure 2 for details). In this way, a specific motif “[I, L, M]^14^-x-P^16^-x-x-x-[F, W]^20^” for the 3074-targeted epitope was derived from the 3D structure of the mAb-viral peptide complex shown in Figure 1A. The corresponding structures for 2557 and 268-D are shown for comparison.

Our prior work demonstrated that the epitope motifs derived by the above method are sensitive and specific predictors of *in vitro* neutralization of a diverse set of viruses [30]. The sequence motif derived from the crystal structure of the Ab:antigen complex for the neutralization epitope targeted by mAb 3074 is unique from the other six motifs (Table 1), therefore we can infer that this motif is a unique signature motif for the neutralization epitope targeted by mAb 3074. A similar analysis was performed for all the seven mAbs we considered with available V3:mAb crystallographic structures to identify a library of unique signature motifs for the neutralization epitopes targeted by all seven anti-V3 mAbs (Table 1).

Overall, the motifs for the Abs are diverse and overlap along the V3 loop crown sequence (Table 1). 2219 and 2557 only engage side chains in the N- and C-terminal strands and are not restricted by amino acid substitutions in the V3 crown β-turn. 268-D, 537-10D, 447-52D, 3791 and 3074, on the other hand, all engage the β-turn, with 3074 specifically restricted by the Pro at position 16 and the others mostly restricted by the Q/R at position 18. Interestingly, only the motifs for 447, 537 and 268 are mutually exclusive with another mAb neutralization epitope (3791) since the former requires an Arg (R) at position 18 of the V3 loop and the latter requires a Gln (Q) at the same position. The other motifs may occur simultaneously in the same V3 loop. The motifs for neutralization epitopes targeted by 2219 and 2557 differ by only one position, despite the fact that these mAbs derive from subjects infected with viruses belonging to different subtypes.

We estimated the global conservation of the seven different neutralization epitopes by searching for the rate of occurrence of their motifs in each subtype in the LANL HIV Sequence Database (http://hiv.lanl.gov), weighting the percent occurrence by each subtype's proportional contribution to circulating HIV strains actually in the human population as estimated by the Hemelaar et al. WHO study, and adding up the total (Table 2 and Methods). mAb 3074's epitope is found in 87% of circulating HIV-1 strains infecting humans, with a significant rate of occurrence in strains from all the major subtypes. mAb 3791 is largely subtype C restricted, but because this subtype represents 50% of the disease pandemic, the neutralization epitope targeted by 3791 is estimated to be conserved in 63% of circulating strains worldwide. mAbs 2219, a subtype B derived mAb, and 2557, a non-subtype B derived mAb, are conserved in 56% and 52% of circulating strains worldwide, and are relatively evenly conserved across the major subtypes. Interestingly, the neutralization epitope 447-52D—perhaps the most cited anti-V3 mAb in the literature—is estimated to be present in only 11% of circulating strains and is highly restricted to subtype B. Finally, the neutralization epitopes targeted by 537-10D and 268-D follow a similar pattern to that for 447-52D: less than 10% conservation in circulating worldwide strains, and predominantly appearing in subtype B strains.

**Table 2:** Fraction of Circulating HIV Strains Worldwide That Contain the Sequence Motifs for anti-V3 mAb Epitopes.

mAb	Subtype A (12%)	Subtype B (10%)	Subtype C (50%)	Subtype D (3%)	Subtype G (6%)	Subtype CRF01_AE (5%)	Subtype CRF02_AG (5%)	Total (100%)
3074	11	8	48	1	3	4	5	87
3791	7	0	45	0	1	1	3	63
2219	6	7	30	0	4	0	3	56
2557	6	7	27	0	4	0	3	52
447-52D	1	7	0	1	0	1	0	11
537-10D	0	6	0	1	0	1	0	9
268-D	0	4	0	0	0	0	0	5

The column headings for each of the subtypes displays the subtype name and, in parentheses, the percentage of the whole set of worldwide circulating viruses that belong to that subtype. Each cell shows the percentage of worldwide viruses, all of that subtype, that contain the sequence motif targeted by the indicated mAb of that row: thus “11%” for 3074 and subtype A indicates that 11% of the 12% of worldwide viruses that are subtype A contain the 3074 targeted epitope. Each cell in the Total column indicates the percentage of worldwide circulating viruses that contain the signature motif of the epitope targeted by the indicated mAb of that row.

**Figure 3:**
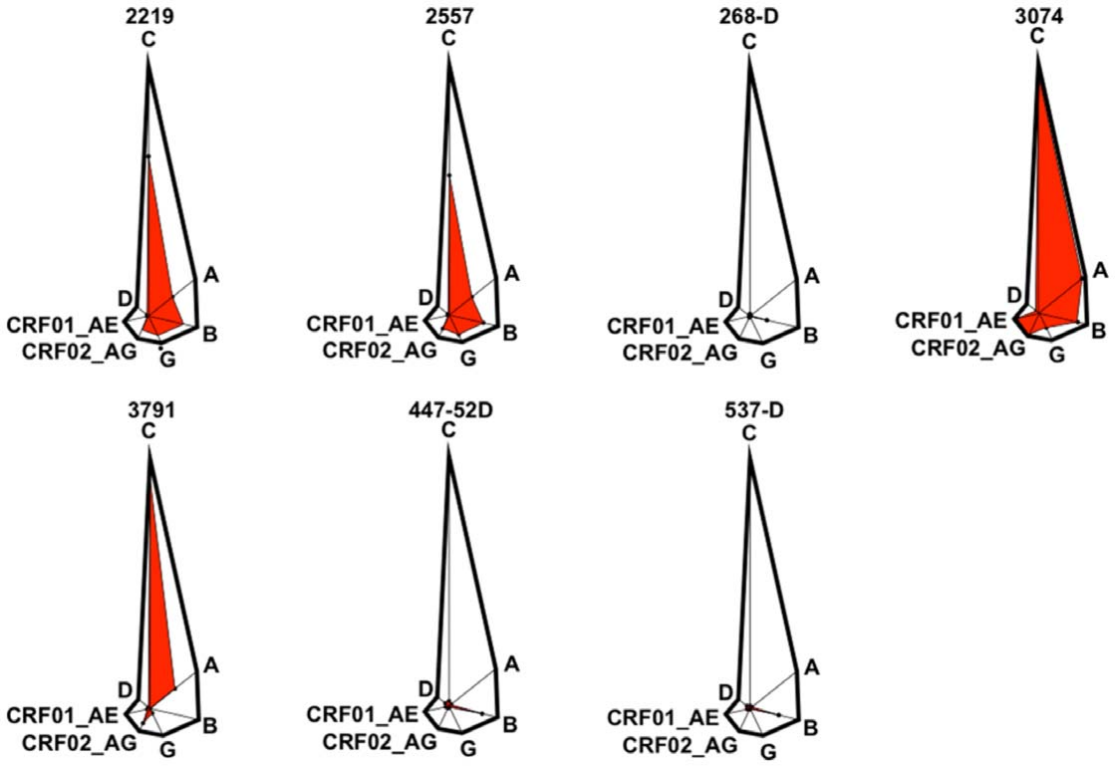
Radar graphs illustrating the data on the global cross-HIV-1-strain conservation neutralization epitopes targeted by mAbs. The mAb is indicated in black bold letters above its graph. Each radar graph is a polygon, each point of which is representative of each subtype and each axis of which is proportional to the fraction of that subtype in the whole set of circulating worldwide strains. Thus the point labeled “C” is subtype C and its axis from the center of the polygon is as long as all the other axes combined, because subtype C represents half (50%) of the worldwide population of viruses. The percentage of each subtype that contains the neutralization epitope targeted by the mAb is plotted on each axis. Connecting each one of these plotted points traces out an inner polygon colored red that is visually representative of the population of circulating viruses that contain the sequence motif for the neutralization epitope targeted by that mAb. Thus, if most of the area enclosed by the polygon is colored red, most of the circulating HIV-1 viruses in the human population worldwide contain the epitope targeted by the indicated mAb in their sequences.

While some predicted anti-V3 loop mAb neutralization epitopes appear to be largely restricted to a single subtype (268, 447-52D to subtype B, for example), several are evenly conserved across all major subtypes (3074, 2219, 2557; Figure 3). Indeed, although 3791's predicted neutralization epitope is largely restricted to subtype C, its overall prevalence (63%) is greater than that of 2219 (56%) or 2557 (52%), whose epitopes are distributed more evenly across the subtypes.

These subtype distribution patterns correlate with recent studies measuring the neutralization of viruses from different subtypes by these mAbs. Although it is difficult to extrapolate the diversity of less than 100 viruses tested in neutralization assays to the diversity of the 10 s of thousands of viruses circulating in the world, the subtype distribution of our informatics assessment clearly correlates with the subtype distribution of the in vitro tests (Table 3). For example, in the in vitro panel of viruses, mostly subtype B viruses are neutralized by mAb 447-52D and this is concordant with the appearance of its cognate epitope mostly in subtype B viruses worldwide.

**Table 3:** Fraction of HIV-1 Pseudoviruses of five subtypes Neutralized by anti-V3 mABs.

mAb	Subtype A (11 psVs)	Subtype B (51 psVs)	Subtype C (24 psVs)	Subtype D (10 psVs)	Subtype CRF02_AG (2 psVs)
3074	18	22	46	10	100
2219	18	49	29	10	0
2557	18	47	21	10	100
447-52D	9	47	4	10	0

Summary of neutralization breadth of individual mAbs from previously published experiments. The column headings for each of the subtypes displays the subtype name and the number of HIV-1 pseudoviruses of that subtype tested for neutralization by the indicated anti-V3 mAbs. Each cell shows the percentage of tested HIV-1 pseudoviruses which are neutralized by the indicated mAb of that row. The *in vitro* neutralization data from Hioe et al. [35] was summarized in this table.

## Discussion

The conservation of different predicted neutralization epitopes targeted by anti-V3 loop antibodies across circulating HIV-1 strains varies widely according to this study, but some highly conserved individual neutralization epitopes are indeed present in this immunodominant, sequence variable region. Epitope conservation ranged from <5% of known HIV viruses for the motif recognized by mAbs such as 268 to 87% for the motif recognized by mAbs such as 3074 (Table 2). The result for mAb 447-52D mirrors an earlier study, but in this updated version, the estimate of 2219 conservation is higher than in the previous study [30]. The relative conservation patterns estimated in this study via informatics are consistent with recent *in vitro* neutralization studies showing that 2219, 2557 and 3074 neutralize a large fraction of diverse viruses carrying pseudotyped envelopes from HIV subtypes A, B, C and D [35]. This means that testing a mAb against a diverse panel of viruses or defining the epitope by our method might independently assess the disease-specific breadth of a mAb at low resolution. Both estimates should be concordant in terms of subtype breadth, as they are for these mAbs, for either to be a reliable as a “broad neutralization score” for an mAb or an epitope: indeed, the concordant combination of the two connects molecular *in vitro* neutralization results using a small viral panel with worldwide clinical bioinformatics recordings of HIV-1 antigenic diversity.

The observed conservation of 3074, 2219 and 2557 epitopes despite their location in a sequence variable loop is also consistent with findings that mAbs targeting compound quaternary neutralizing epitopes composed of V2 and V3 can neutralize pseudoviruses carrying the envelopes of primary isolates from diverse subtypes[36]. Conserved epitopes are thus hidden in the immunogenic sequence variable loops of gp120, but the challenges are finding them, assessing the magnitude of their conservation and exploiting them in immunogen design. This study provides a tool for addressing the second of these three challenges.

Our results show that clusters of circulating viruses defined by the presence of a particular mAb epitope do not consistently correlate with the clusters of viruses defined phylogenetically by subtype (Figure 3). This is not a surprising finding given that the whole viral genomic sequence determines the subtype while as little as one amino acid in the sequence may define an epitope. However, the demonstration of this divergence in our results calls into question the use of the term “broadly neutralizing” to describe certain mAbs, when the use of the term is based solely on the ability of the mAb to neutralize multiple viral subtypes. The conceptual flaw in this assumption is easily seen via *reductio ad absurdum*: a mAb targeting an epitope present in only 5 viruses each from a different subtype would be considered extremely broad in terms of its breadth across subtypes by the prevailing definition, but would in reality have essentially no neutralization/antigenicity range among globally circulating HIV viruses. In practice, our results show that the assumption of antigen-type/genotype equality is somewhat unreliable: mAbs 2219, 2557 and 3074, whose epitopes appear independently of subtype, also occur quite widely in circulating viruses. However, the assumption that neutralizing only one subtype equates with disease irrelevance should be made with caution: 3791 is almost completely subtype C restricted, but because subtype C is such a large proportion of circulating viruses, protection against this epitope may be highly disease relevant in certain epidemic areas and globally would represent 63% of circulating viruses.

Specifically, our results call into question any extrapolation of results with 447-52D to characterization of the overall antigenicity of the V3 loop. 447-52D is commonly used in the literature to represent the activity of anti-V3 mAbs, but our results show that its neutralization epitope is only present in 11% of circulating viruses and mostly in subtype B, and that it is more of an outlier than a representative mAb (Figure 3). Indeed, our results and recent studies of 447-52D neutralization of diverse panels of viruses show that it is subtype B restricted [17], [35]. Thus, conclusions based on observations with 447-52D, which are the most numerous in much of the literature invoking anti-V3 mAbs, are really only applicable to HIV phenomenology in North America and Europe and ignore the significant contribution of subtype C to the pandemic, estimated at half of all circulating viruses. In reality, our study shows that the distributions of epitopes targeted by individual anti-V3 mAbs are quite variable, so no single mAb is representative of V3 loop antigenicity.

The sequence motifs for the neutralization epitopes targeted by mAbs 2219 and 2557 were found to be very similar, despite the fact that they were derived from the cells of two different individuals, infected with different virus subtypes (B and CRF02_AG, respectively) from two different geographic regions (North America and Africa, respectively) [24], [29]. Thus, despite originating from infections with very different viruses in very different global locations, the neutralization epitopes of these two mAbs are very similar both in structure and in distribution.

From a technical point of view, while our studies have shown that the signature motifs are highly accurate, they are dependent on our as-yet-imperfect method for defining which amino acid can substitute at the key sites in the motif based on energetic/modeling studies on the crystallographic structural data, without accounting for the insertions and deletions that rarely occur in the V3 loop. Studies correlating these modeling approaches with additional pseudovirus neutralization measurements may improve the precision of the motifs marginally.

These results have several implications for HIV vaccine design. This study only reports results based on the physical presence of antibody-targeted epitopes in HIV viruses. The physical presence of an epitope in an HIV virus does not equate to neutralization of the virus or immunogenicity of the epitope. Epitopes in gp120 are well known to be differentially masked by glycans or occlusion by other protein regions [37]–[39]. This is true for V3 loop neutralization epitopes as well [40]. The finding reported in this study is a crucial step in attempting to characterize masking: viruses that can be ascertained to contain epitopes by the methods reported here that are not neutralized by the cognate mAbs in vitro necessarily mask those epitopes. Thus, by combining our approach with in vitro monoclonal antibody-mediated neutralization assays, masking of specific mAb targeted epitopes can be precisely characterized. Epitope masking is expected to be the major factor reducing the practical cross-strain neutralization value of any given conserved anti-variable-loop epitope from its theoretical maximal cross-strain reactivity to its practical cross-strain reactivity expected for a vaccine.

Other factors influence neutralization even when the epitope is present and not masked. For example, dynamic tertiary folding of the V3 loop in a particular strain may disrupt the epitope despite presence of the key side chains. This phenomenon may manifest itself as variable affinity of anti-V3 mAb even for viruses in which the V3 loop is exposed and accessible. Nevertheless, there is an extensive literature showing that anti-V3 mAbs can neutralize up to 55% of primary isolates [28], [41]–[43]. Thus, neutralization epitopes in the V3 loop are not “cryptic” in all viruses, and V3 masking is not an all-or-none phenomenon [44]. Moreover, it has previously been shown that the exposure of V3 can be induced and or enhanced by various gp120 ligands such as CD4 [45], [46] and anti-CD4bs [47] and anti-V2 Abs [48]. Targeting certain conserved variable loop epitopes, such as 3074, 2219, 2557 and 3791, for immunogen design takes advantage of the immunodominance of these regions, and the resulting immune serum might well be broadly, not narrowly, effective even in the presence of masking. Immunogenicity of an epitope is a function of its physical presence in the virus, how masked it is in the virus, and human immunologic factors. With this study, the first of these factors can be controlled so that studies to characterize masking and, in turn, immunogenicity of specific epitopes can be pursued with observational clarity.

Our estimates of the extent and epidemiological boundaries of the conservation of mAb-defined neutralization epitopes may be useful in polyvalent HIV neutralizing antibody vaccine design, as they provide a means to “see through” the diversity of molecular configurations of the HIV envelope present in circulating viruses. The method we have described provides a means to precisely observe the occurrence of the 3074 epitope, for example, within any envelope molecular configuration, and indeed within all configurations at the same time (Figure 3). “Reverse vaccinology” strategies start from interesting neutralizing mAbs and work backwards to design immunogens mimicking the neutralization epitopes targeted by the mAbs. The selection of which variable loop targeted mAb to mimic using these strategies is now simplified by this approach: mAbs such as 3074 targeting highly conserved epitopes are now easily distinguishable from less prevalent mAbs such as 447-52D (Figure 3) to enhance the neutralization information emerging from testing of the mAbs against small panels of viruses. The motifs we have described can be used to precisely partition viruses, separating those that contain the neutralization epitope from those that do not contain the neutralization epitope. The latter, of course, are unlikely to be neutralized by the antibody. Immunogens designed to elicit specific antibodies can thus be combined into a vaccine on the basis of minimal or maximal overlap of sets of circulating viruses. For example, two immunogens designed to elicit 447-52D and 3791 antibodies respectively would target two completely different sets of circulating strains (in subtypes B and C respectively), so their effects may be complementary. Conversely, two immunogens designed to elicit 2219 and 2557 would reinforce each other in targeting the same set of circulating viruses. Multiple rational combinations are possible using the motifs.

Finally, although exploiting the epitope characterization technology described here to elicit a specific antibody response in animals is a challenge, recent DNA-prime and V3 loop protein boost immunofocusing strategies were successful in exploiting the immunodominance of the V3 region and stimulating robust cross-strain neutralizing animal serum responses targeted to the V3 loop [49], [50]. This suggests that the natural immunogenicity of variable loop regions is indeed a window of opportunity in HIV vaccine design, and the global conservation approach described here for V3 loop neutralization epitopes may be immediately useful for solving the “broad neutralization” problem of these regions by identifying promising variable loop targeted mAb, suchas 2219, 2557, 3791 and 3074, for “reverse-vaccinology” approaches.
